# Enhancement of Perivascular Spaces in 7 T MR Image using Haar Transform of Non-local Cubes and Block-matching Filtering

**DOI:** 10.1038/s41598-017-09336-5

**Published:** 2017-08-17

**Authors:** Yingkun Hou, Sang Hyun Park, Qian Wang, Jun Zhang, Xiaopeng Zong, Weili Lin, Dinggang Shen

**Affiliations:** 1 School of Information Science and Technology, Taishan University, Taian, 271000 China; 2 Department of Robotics Engineering, Daegu Gyeongbuk Institute of Science and Technology, Daegu, 42988 South Korea; 3 Med-X Research Institute, School of Biomedical Engineering, Shanghai Jiao Tong University, Shanghai, 200030 China; 4 Department of Radiology and Biomedical Research Imaging Center, University of North Carolina at Chapel Hill, Chapel Hill, NC 27599 USA; 5 Department of Brain and Cognitive Engineering, Korea University, Seoul, 02841 South Korea

## Abstract

Perivascular spaces (PVSs) in brain have a close relationship with typical neurological diseases. The quantitative studies of PVSs are meaningful *but* usually difficult, due to their thin and weak signals and also background noise in the 7 T brain magnetic resonance images (MRI). To clearly distinguish the PVSs in the 7 T MRI, we propose a novel PVS enhancement method based on the Haar transform of non-local cubes. Specifically, we extract a certain number of cubes from a small neighbor to form a cube group, and then perform Haar transform on each cube group. The Haar transform coefficients are processed using a nonlinear function to amplify the weak signals relevant to the PVSs and to suppress the noise. The enhanced image is reconstructed using the inverse Haar transform of the processed coefficients. Finally, we perform a block-matching 4D filtering on the enhanced image to further remove any remaining noise, and thus obtain an enhanced and denoised 7 T MRI for PVS segmentation. We apply two existing methods to complete PVS segmentation, i.e., (1) vesselness-thresholding and (2) random forest classification. The experimental results show that the PVS segmentation performances can be significantly improved by using the enhanced and denoised 7 T MRI.

## Introduction

**Figure 1:**
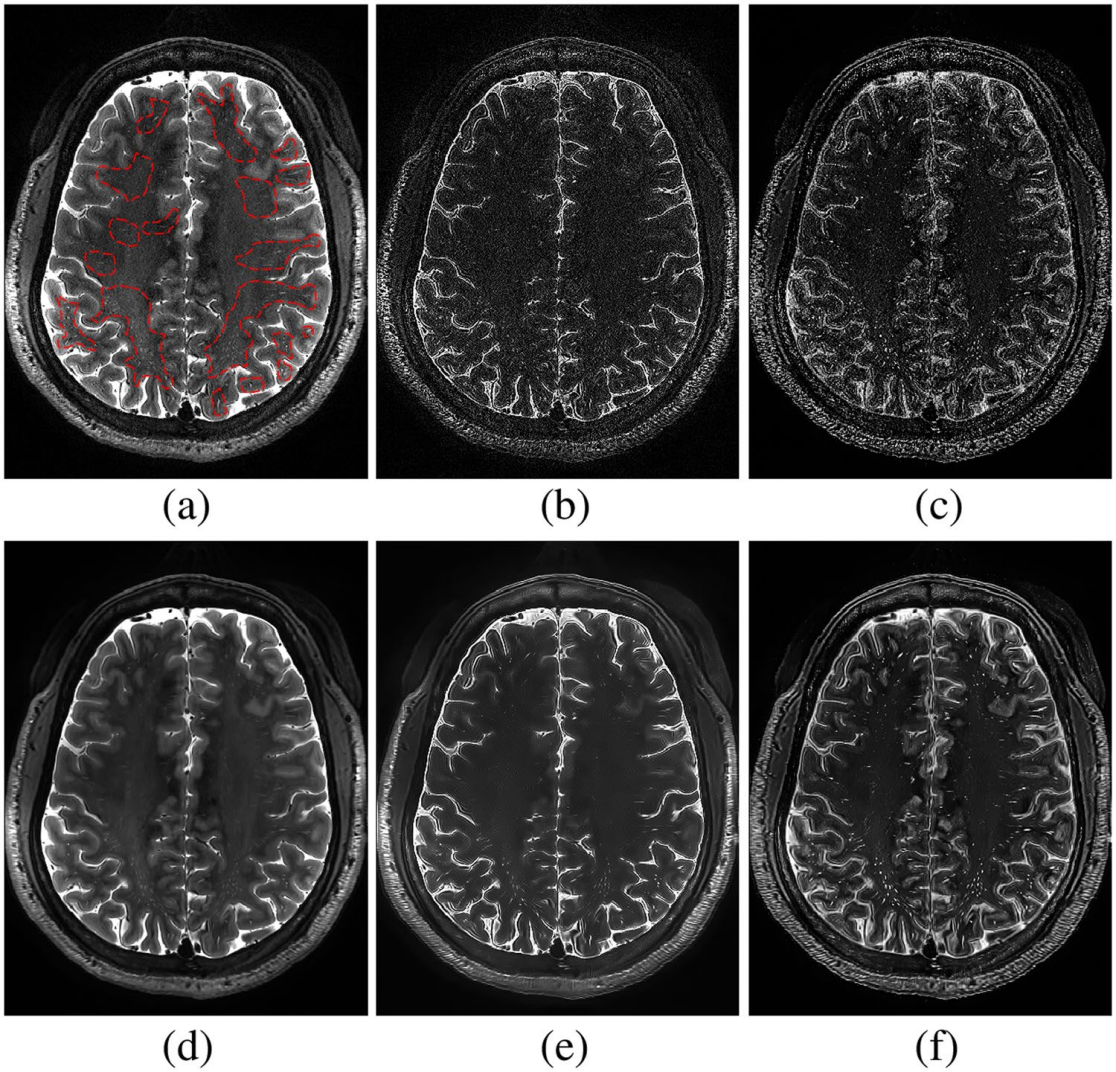
(**a**) Illustration of PVSs in a 7 T MR image. Those relatively bright voxels within the red dotted contours are the PVSs. (**b**) Enhanced image by using spatial correlation filtering; and (**c**) enhanced image by using our proposed method. (**d**–**f**) Show the denoised images of (**a**–**c**), respectively. For these three cases, the BM4D method uses the same parameters to denoise the images.

The perivascular space (PVS) is the normal anatomical structure filled with cerebrospinal fluid (CSF) in the nerve system. It was initially identified by a German pathologist, *R. Virchow*, and a French biologist, *C.P. Robin*, more than a century ago. The PVS, which is also known as the perivascular lymphatic space, is related to brain physiological and immune functions^1–4^. For example, the diameter of PVSs is typically less than 2mm in all age groups of healthy people. The abnormal enlargement of the diameter *or* the increase of the PVS number is related to aging^5–7^, cognitive degeneration^8^, and vessel diseases^6, 9, 10^. Although PVS has drawn more interest in recent years, the traditional 1.5 T and 3 T magnetic resonance (MR) scanners can hardly capture the thin structures of PVSs precisely. The new-generation 7 T MR scanner, on the contrary, owns much better capability of rendering PVSs^10^. However, it is still difficult to clearly distinguish the thin PVSs from the noisy background in the 7 T MR images. Figure 1(a) shows the examples of PVSs in a 7 T MR image, where those relatively bright voxels within the red dotted contours are the PVSs. We can see that the PVSs are the thin structures with weak signals in the 7 T MR image.

There are several reports that focus on the identification or segmentation of PVSs. For example, Wuerfel *et al*.^11^ developed a semi-automatic software that can adjust the intensity threshold for segmenting PVSs by optimizing a predefined PVS model through Markov Chain Monte Carlo. Uchiyama *et al*.^12^ used the white top-hat transform to enhance the tubular structures of PVSs, then extracted them according to intensity thresholding, and then finally identified PVSs using the geometric properties. Ramirez *et al*.^13, 14^ proposed to segment PVSs in a semi-automatic way by determining the intensity thresholds from T2-weighted and proton density (PD) images adaptively. Wang *et al*.^15^ also proposed a semi-automatic method that can adaptively adjust the intensity threshold by using gamma correction and linear mapping. Recently, Park *et al*.^16^ proposed a learning-based automatic segmentation method by training a sequence of random forest classifiers with the orientation-normalized 3D Haar features. Then, the segmentation is attained through the sequential classifiers, followed by vesselness thresholding. Although these existing methods can effectively use various characteristics of PVSs, the segmentation performance is still limited due to image quality. In particular, it is difficult to extract informative features of the thin and weak PVS structures from the noisy background.

Accordingly, it is necessary to enhance image quality to effectively distinguish the PVS structures. A common image enhancement is through spatial correlation filtering, which performs convolution operation on every voxel. However, the convolution operation using a single kernel cannot adaptively amplify the PVS signals and also, at the same time, suppress the noises (see Fig. 1(b)). To effectively enhance the specific PVS-like structures, Uchiyama *et al*.^12^ incorporated a white top-hat transform method, which is relatively less sensitive to both image noise and inhomogeneous intensity condition. However, since the white top-hat transform is also based on a simple morphological filtering, it cannot fully utilize the nearby spatial and appearance information to enhance the PVSs.

**Figure 2:**
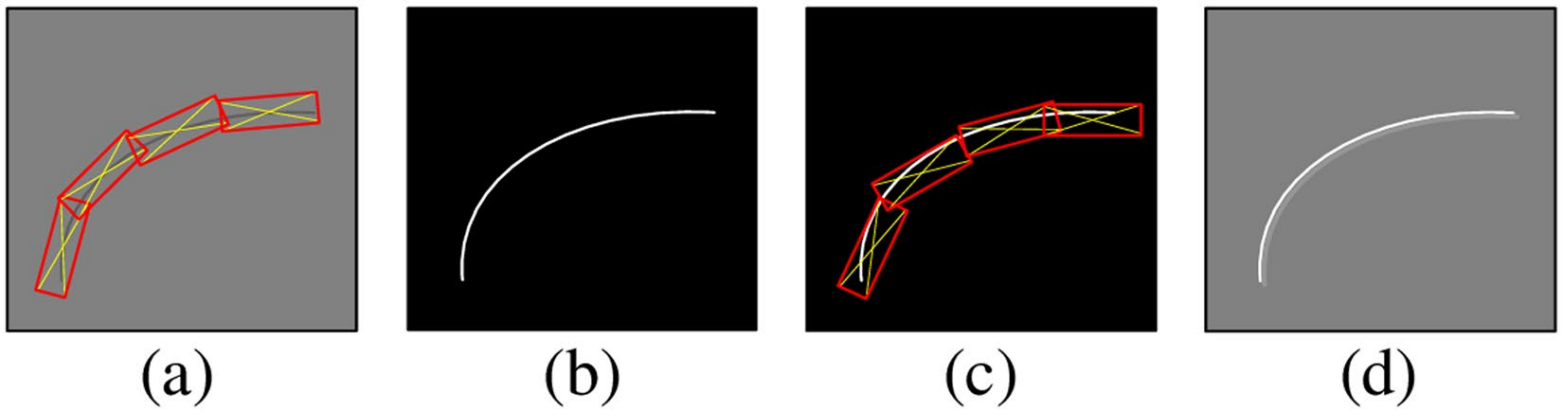
Illustration of halo artifacts by NSCT image enhancement. (**a**) Original image and NSCT forward transform; (**b**) enhanced transform coefficients; (**c**) NSCT inverse transform; and (**d**) enhanced image with halo artifacts.

To address these limitations, it is necessary to apply PVS enhancement and image denoising *prior to* PVS segmentation. Based on the observation that the PVSs are usually presented as thin tubular structures, we can effectively describe the PVSs with the line singularity representation^17, 18^. Traditional orthogonal wavelet transform^19, 20^, as widely used in image processing and singularity detection, only represents the point singularity. Accordingly, a series of redundant wavelets (including Ridgelet^21, 22^, Curvelet^23^, Contourlet^17^, NSCT^18^, etc.) have been developed to capture the line singularity. However, *either* the orthogonal wavelets *or* the redundant wavelets just directly convolute the local image neighborhood by using a certain convolution kernel. Thus, image artifacts are inevitably introduced during the local convolution-based transform. These artifacts may seriously decrease the accuracy of the PVS segmentation. For example, NSCT uses the long and narrow filter banks to achieve the linear singularity representation, thus the inverse transform usually introduces halo artifacts due to enhanced transform coefficients, as shown in Fig. 2. From Fig. 2(c), we can see that those near the enhanced coefficients are usually affected by the enhanced coefficients. Thus, they are likely to also be enhanced during the inverse transform, which introduces the halo artifacts. To this end, the local transform may not be optimal to enhance the PVSs for the subsequent PVS segmentation.

To suppress the artifacts for better image enhancement and denoising, the nonlocal strategy, such as nonlocal-means (NL_means)^24^ and block-matching & 3D filtering (BM3D)^25, 26^, have been developed. These nonlocal methods try to find a number of nonlocal image blocks, which are (1) visually similar to the reference block and (2) located within the nonlocal search range of the reference block. The weighted mean of the nonlocal image blocks is used to remove the noise^24^. In BM3D method, a separable 3D transform on the stacked 3D array of the matched similar blocks (by implementing 2D transform on each 2D image block and then performing 1D transform on the third dimension) is used to obtain the enhanced sparse representation of the image, and finally, by hard-thresholding the transformed coefficients, the reasonable image denoising result can then be achieved^25, 26^. On the other hand, the nonlocal filtering methods have been further extended to address the higher-dimensional problems (BM4D)^27, 28^ in video and 3D medical image data. Although both types of these nonlocal methods and BM3D methods are effective on image denoising, they fail to enhance the PVSs in 7 T MR images since PVSs are tiny structure in the 7 T MR images and thus the matched nonlocal image blocks are highly similar to each other. In this way, PVSs cannot be separated from the background.

In this paper, inspired by the nonlocal image denoising method, we propose a novel PVS enhancement method using the nonlocal line singularity representation. We first extract *K* neighboring cubes from a small neighborhood of the reference cube, according to their top-left corner coordinates. All *K* extracted cubes are formed into a group, upon which we perform 1-D Haar transform. Then, we process the Haar transform coefficients by using a nonlinear mapping function, in order to amplify image details and suppress noise simultaneously. Next, the enhanced *K* neighboring cubes can be reconstructed from the processed Haar transform coefficients. By repeating these steps for reference cubes in the whole image, we can obtain an enhanced 7 T MR image (see Fig. 1(c)). Finally, we apply the existing BM4D method to further denoise the enhanced image, which is referred to as the enhanced and denoised image (see Fig. 1(d)) in this paper. To verify the effectiveness of our proposed image enhancement and denoising method, we use two existing PVS segmentation methods, i.e., (1) vesselness thresholding and (2) random forest learning^16^, to segment PVSs from the original 7 T MR images and also our enhanced and denoised 7 T MR images. The experimental results show that our proposed enhancement and denoising method can significantly improve image quality and potentially help with PVS segmentation.

## Method

**Figure 3:**
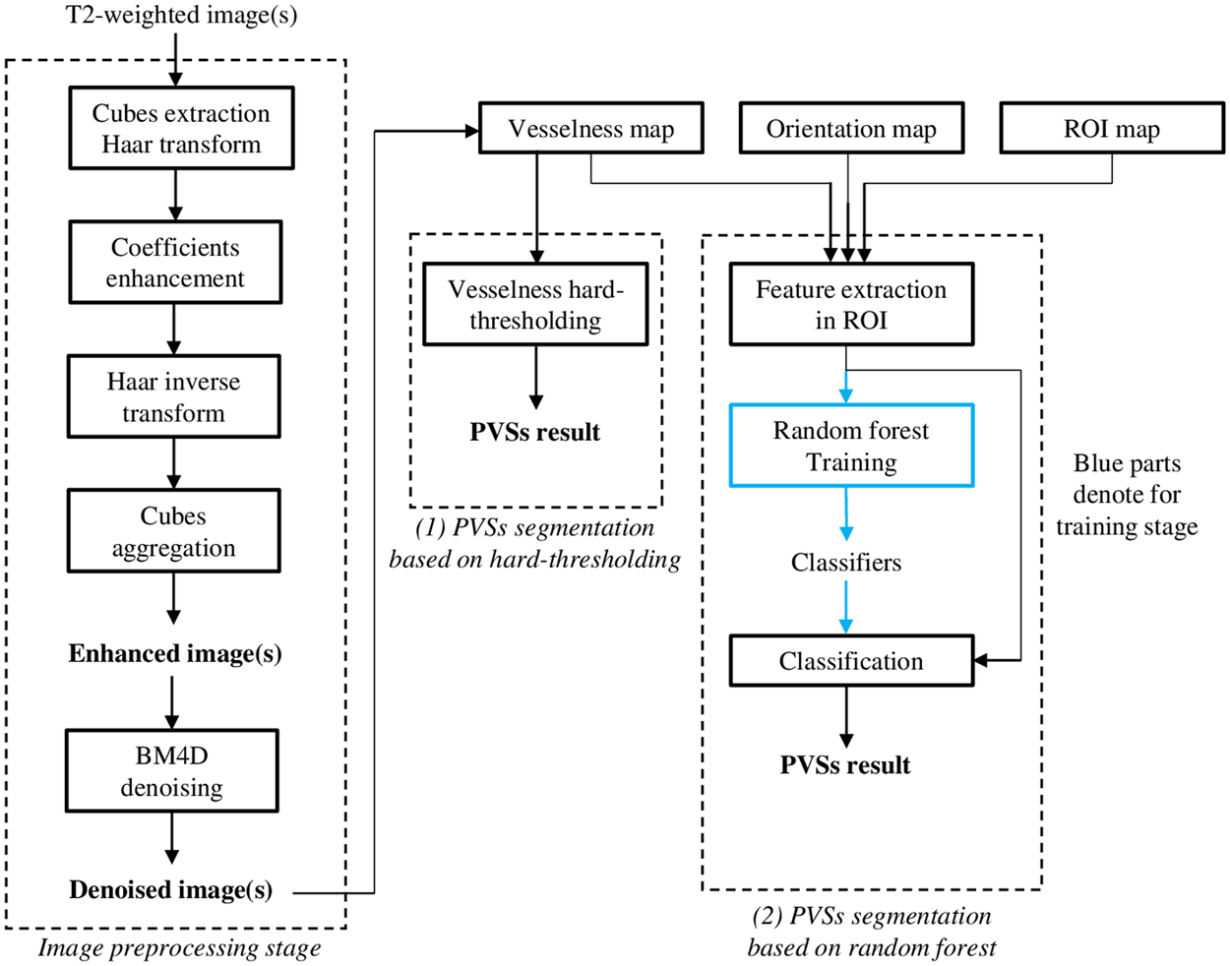
Framework of the proposed image enhancement (left panel) and PVS segmentation algorithm (right panel). After enhancing the image, two existing methods are used to segment the PVSs, i.e., (1) PVS segmentation based on hard-thresholding and (2) PVS segmentation based on random forest.

Our proposed framework for PVS enhancement and segmentation is summarized in Fig. 3. Our PVS enhancement method is mainly based on the Haar transform of nonlocal cubes. In particular, first, we extract *K* neighboring cubes within a small neighborhood (i.e., 3 × 3 × 3) of the center of each reference cube in the 7 T MR image. (Note that, if a cube’s top-left corner location is included in the small neighborhood (i.e., 3 × 3 × 3) of the reference cube, it can be extracted as a neighboring cube.) Then, we perform the Haar transform on the group of all the extracted nonlocal cubes. Note that, after Haar transform, the PVSs can be effectively represented in the transformed subbands. Next, to enhance PVSs, according to the characteristics of PVSs, we further process the transformed coefficients by nonlinear mapping to simultaneously amplify the signals (relevant to PVSs) and also suppress the noise. In this way, the enhanced PVSs can be effectively reconstructed by the inverse Haar transform, given the processed (transformed) coefficients. By reconstructing all reference cubes from the whole 7 T MR image, all reconstructed cubes can be aggregated into a single enhanced image. Finally, we apply the existing BM4D image-denoising method^28^ to further suppress the noise since the enhanced image may still contain noise that can affect PVS segmentation. Note that, we have released our image enhancement software to the public^29^. To prove that our proposed enhancement method is effective for the PVS segmentation, we compare the PVS segmentations by adopting two existing segmentation methods, *i.e*., vesselness thresholding based method and random forest based method, from the original 7 T MR images, the denoised images (by directly applying BM4D on the original 7 T MR images), and our enhanced and denoised images (by our complete method). The evaluation was performed on seventeen 7 T MR images^16^ from healthy volunteers ranging from ages 25 to 37. Informed consents were obtained for both study participation and publication of their images without identification information. The study was reviewed and approved by the institutional reviews board of University of North Carolina and the methods used in this study were carried out in accordance with the approved guidelines. Further details of the experimental setting are described in Section 3.

### Nonlocal Haar Transform

**Figure 4:**
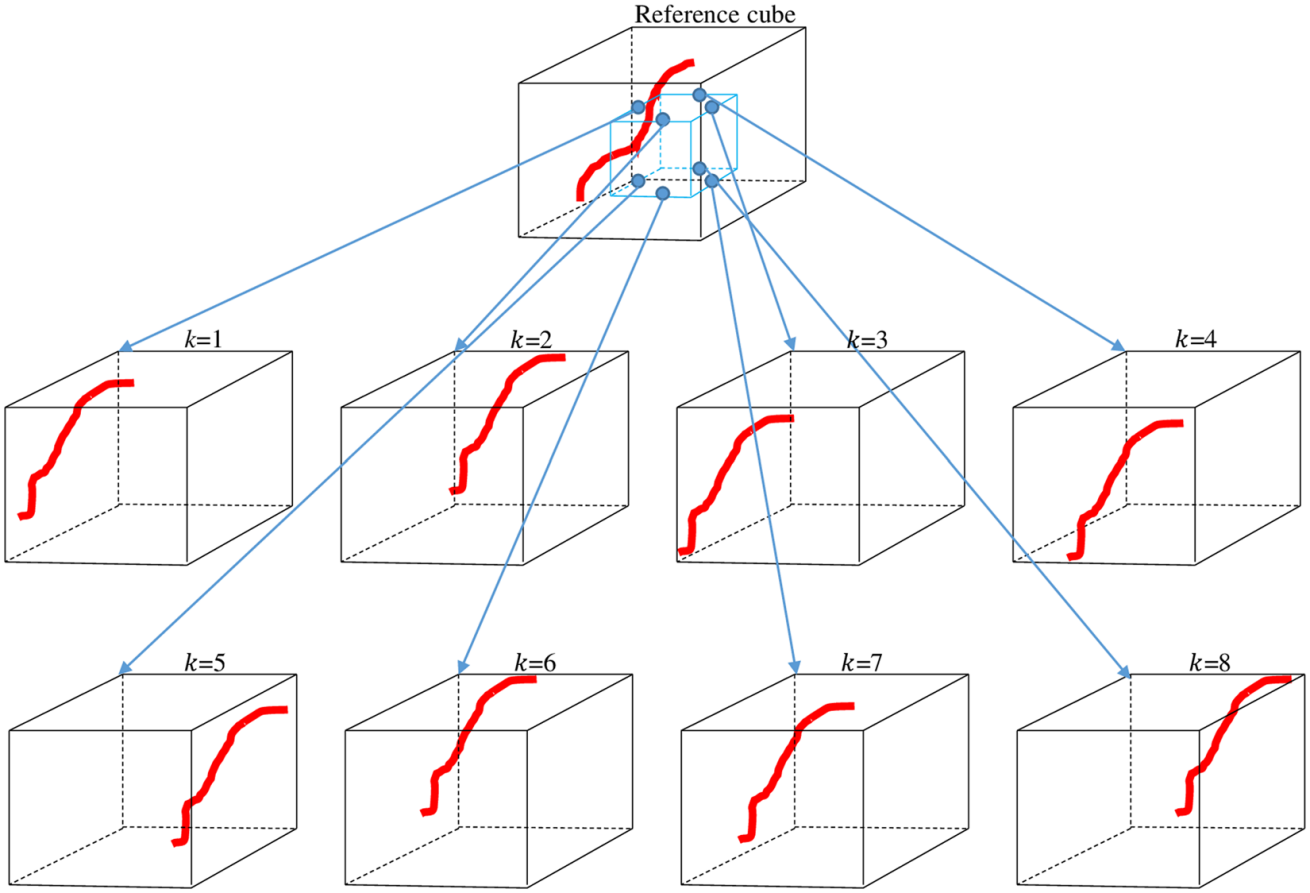
An illustration of the extraction of neighboring cubes (*K* = 8) from the small neighborhood (i.e., small blue block in the top of figure) around the center of the reference cube.

The goal of this step is to enhance the 7 T MR image by better revealing the details of the PVSs. Again, our enhancement strategy is inspired by the nonlocal strategy in the literature, and is particularly implemented through the Haar transform. Specifically, first, we extract *K* (i.e., *K* = 8) neighboring image cubes (with the same size as the reference cube) from a small neighborhood (i.e., 3 × 3 × 3) around the center of the reference cube. Then, all the extracted cubes are formed into a group, denoted as ${{\boldsymbol{C}}}_{{\boldsymbol{E}}}=[{C}_{1}\cdots ,{C}_{i},\cdots ,{C}_{K}]$ where *C*_*i*_ is a 7 × 7 × 7 cube. Figure 4 shows an example of neighboring cube extraction scheme for the case of *K* = 8. We can observe that the patterns of the PVSs in the extracted cubes are slightly mis-aligned with each other and with the reference cube. Thus, the high-order linear operation (i.e., subtraction) between the two extracted cubes will amplify subtle differences between the included PVSs structures and contribute to capturing the PVSs in the extracted neighboring cubes. This motivates us to apply the Haar transform^30^ across all extracted nonlocal cubes, for enhancing the PVSs.

#### Forward Transform

We apply the forward transform to the extracted neighboring cubes, in order to allow them to represent the PVSs in the extracted cubes. Each neighboring cube is denoted as ${C}_{i}\,(i=1,\cdots ,K)$. Taking *K* = 8 in our implementation, we can use the following formulation to perform the forward Haar transform:

1 $${\hat{C}}_{i}=\sum _{j=1}^{K}{\rm{\Psi }}(i,j){C}_{j},\,(i=1,\cdots ,K),$$

where Ψ(*i*, *j*) is the Haar measure at the crossing of the *i*-th row and the *j*-th column of the Haar transform matrix. Specifically, the 8 × 8 Haar transform matrix can be defined as below:

2 $${\rm{\Psi }}=(\begin{array}{llllllll}1/\sqrt{8} & 1/\sqrt{8} & 1/\sqrt{8} & 1/\sqrt{8} & 1/\sqrt{8} & 1/\sqrt{8} & 1/\sqrt{8} & 1/\sqrt{8}\\ 1/\sqrt{8} & 1/\sqrt{8} & 1/\sqrt{8} & 1/\sqrt{8} & -1/\sqrt{8} & -1/\sqrt{8} & -1/\sqrt{8} & -1/\sqrt{8}\\ 1/\sqrt{4} & 1/\sqrt{4} & -1/\sqrt{4} & -1/\sqrt{4} & 0 & 0 & 0 & 0\\ 0 & 0 & 0 & 0 & 1/\sqrt{4} & 1/\sqrt{4} & -1/\sqrt{4} & -1/\sqrt{4}\\ 1/\sqrt{2} & -1/\sqrt{2} & 0 & 0 & 0 & 0 & 0 & 0\\ 0 & 0 & 1/\sqrt{2} & -1/\sqrt{2} & 0 & 0 & 0 & 0\\ 0 & 0 & 0 & 0 & 1/\sqrt{2} & -1/\sqrt{2} & 0 & 0\\ 0 & 0 & 0 & 0 & 0 & 0 & 1/\sqrt{2} & -1/\sqrt{2}\end{array}).$$

By applying the above Haar transform matrix, all neighboring cubes can be decomposed into 8 subbands. The first subband ${\hat{C}}_{1}$ is the weighted average of all neighboring cubes, while the detailed differences among the cubes are preserved in the rest subbands.

#### Inverse Transform

The Haar transform matrix defined in Eq. (2) is invertible. Thus, we can reconstruct the enhanced *K* neighboring cubes from the computed coefficients of subbands by using the following inverse transform:

3 $${\widehat{\hat{C}}}_{i}=\sum _{j=1}^{K}{{\rm{\Psi }}}^{-1}(i,j){\hat{C}}_{j},\,(i=1,\cdots ,K),$$

where Ψ^−1^ denotes the inverse matrix of Ψ.

After reconstructing all enhanced *K* neighboring cubes in the whole 7 T MR image, we can put them back into their original locations to build a final reconstructed 7 T MR image. Note that the extracted *K* neighboring cubes are overlapping with each other, which indicates that their corresponding reconstructed cubes are also overlapping. In this way, we average the signals of the same location from the neighboring reconstructed cubes to obtain the final reconstructed 7 T MR image.

### Image Enhancement

Image enhancement can be achieved by amplifying the transformed coefficients related to the signals (i.e. PVSs structures). Thus, the appropriate representation of signals is critical to enhance the 7 T MR image. Note that, if the signals are not well-represented, signals and background noise can both be amplified, making it difficult to separate them. Our proposed nonlocal Haar transform can effectively address this problem by simultaneously amplifying the signals (related to PVSs) and suppressing the background noise. Moreover, the artifacts can be alleviated in our method by avoiding the use of large-size filter banks, which were often used in some local transforms (i.e., Contourlet^17^, NSCT^18^, etc.) to represent line singularity.

The proposed image enhancement algorithm can be summarized below:

1. Extract *K* neighboring cubes for each reference cube.
2. Implement the forward Haar transform on the stack of all the extracted *K* neighboring cubes according to Eq. (1).
3. Amplify the details and suppress the background noise by mapping the transform coefficients according to Eq. (4) below:
  4 $${c}_{{\rm{En}}}=\{\begin{array}{lll}{c}_{{\rm{H}}},\, & if & |{c}_{{\rm{H}}}| > {T}_{1}\,\\ {\gamma }_{1}{c}_{{\rm{H}}}, & if & {T}_{2}\le |{c}_{{\rm{H}}}|\le {T}_{1}\\ {\gamma }_{2}{c}_{{\rm{H}}} & if & {T}_{3} < |{c}_{{\rm{H}}}| < \,{T}_{2}\\ 0 & if & |{c}_{{\rm{H}}}| < {T}_{3}\end{array},$$
  where *c*_H_ is the computed Haar transform coefficient, *c*_En_ is the enhanced Haar transform coefficient, and *T*_1_, *T*_2_ and *T*_3_ are the three thresholds used to determine whether the particular Haar transform coefficient corresponds to the common image edge, weak image edge, or noise. Here, we just use the gain factors *γ*_1_ and *γ*_2_ to amplify the weak edges. All these five parameters (*T*_1_, *T*_2_, *T*_3_, *γ*_1_, *γ*_2_) are the empirical values. Specifically, we set *T*_1_ and *T*_3_ so that the Haar transform coefficients of PVSs are included between *T*_1_ and *T*_3_ and then investigate *T*_2_ within *T*_1_ and *T*_3_ to distinguish the common image edges and the ambiguous structures. Since the most common edges with the Haar transform coefficients within *T*_1_ and *T*_2_ are PVSs, the gain factors *γ*_1_ is determined by a relatively large value so that the enhanced edges are clearly shown. On the other hand, since it is difficult to tell the difference between coefficients of very weak edges and noise, we use a relatively small gain factor *γ*_2_ (*i.e*., half of *γ*_1_) to amplify those small magnitude coefficients and potentially also enhance the very weak edges.
4. Implement the inverse Haar transform to enhance those *K* extracted neighboring cubes according to Eq. (3).
5. Aggregate all the enhanced *K* neighboring cubes of each reference cube according to the way described in the end of Section 2.1, and finally obtain an entire enhanced 7 T MR image.

### Further Image Denoising

**Figure 5:**
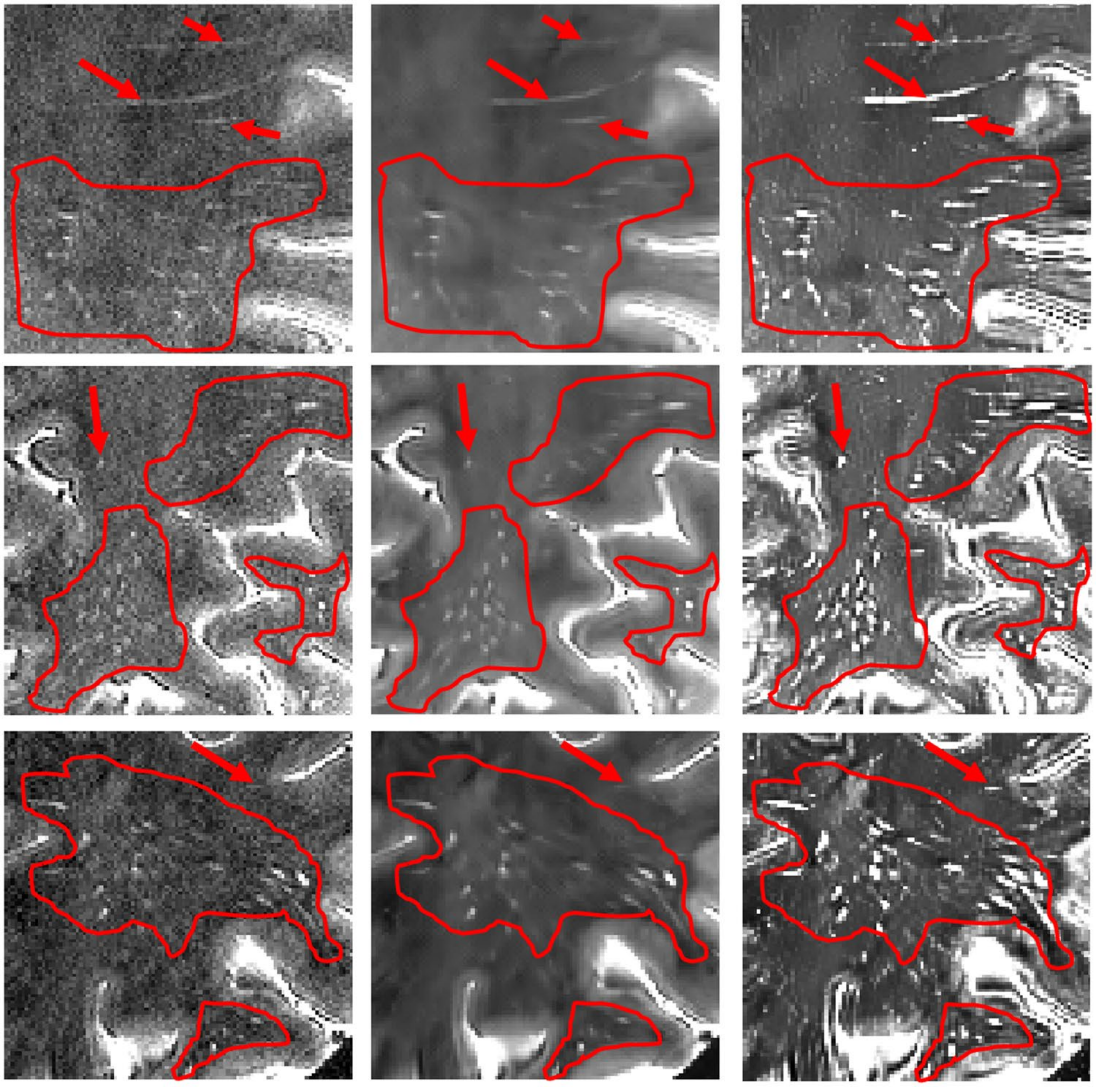
Zoom-in views of the original image (left), the denoised image obtained directly from the original image (middle), and the denoised image from our enhanced image by our complete method (right). Please compare PVSs within contours or pointed by arrows.

By using the above-proposed processing steps, we can obtain the enhanced 7 T MR image. However, because we use a small threshold *γ*_2_ to distinguish between the very weak edges and the noise, the enhanced image may still contain some noise. To address this issue, we further use the BM4D method^28^ to denoise the enhanced image. As briefly mentioned above, BM4D is a state-of-the-art 3D image denoising method. It distinguishes the cubes that are the most similar by cube-matching within a noisy 3D image, and then it stacks similar cubes into a 4D array, and finally performs the separable 4D transform on the stacked 4D array (i.e., applying the separable 3D transform to each cube, and then 1D transform on the fourth dimension). Using this method, noise coefficients can be effectively separated from the signal coefficients in order to extract a reasonably denoised image. As shown in Fig. 1, if denoising is directly performed on the original 7 T MR image, many PVSs are smoothed, but noise can still be effectively suppressed. On the other hand, the PVSs are effectively enhanced while the noise is removed when applying our enhancement and denoising method. Figure 5 further zooms in the original 7 T MR image, the denoised image from the original 7 T MR image, and the denoised image from our enhanced image (with our complete method).

### PVS Segmentation on enhanced images

After enhancing and denoising the 7 T MR images by our above-proposed method, we turn to segment the PVSs from our enhanced and denoised images. In particular, we adopt two existing PVS segmentation methods, as briefly introduced below.

#### Vesselness Based Segmentation

**Figure 6:**
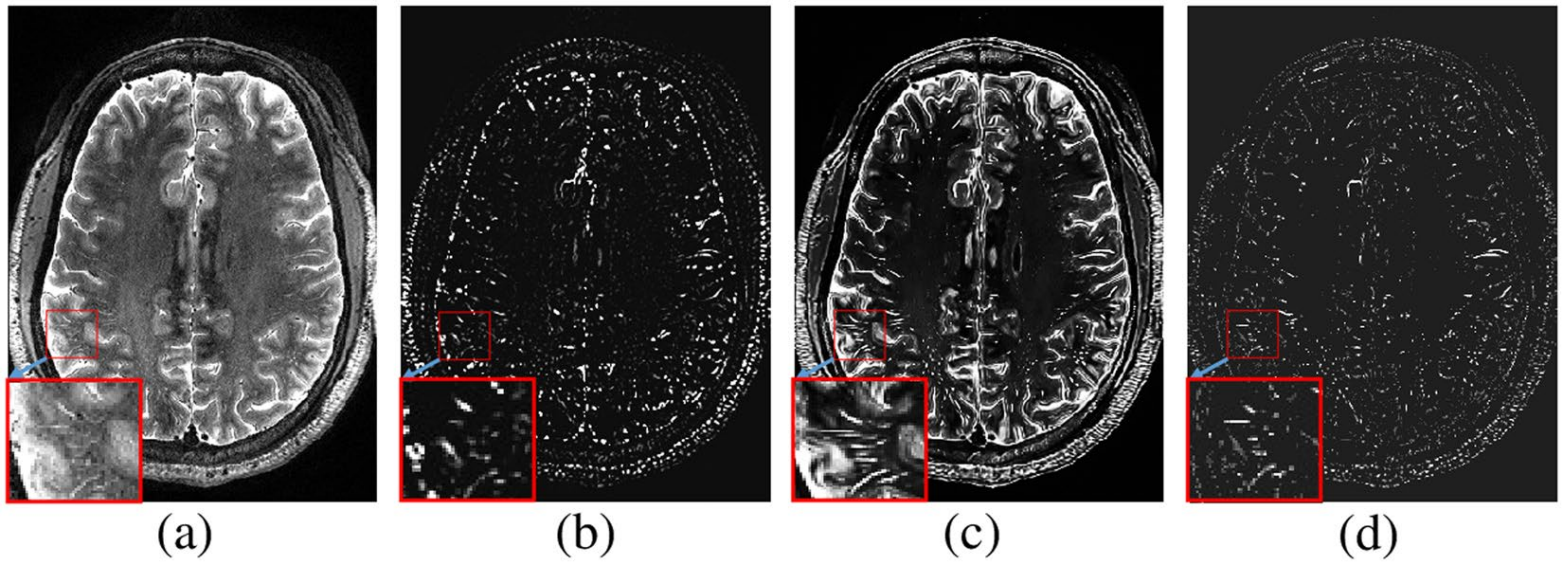
Vesselness estimation on the original image and our enhanced and denoised image. (**a**) Original image, (**b**) estimated vesselness map from (**a**,**c**) our enhanced and denoised image, and (**d**) estimated vesselness map from (**c**).

Vesselness thresholding method is one of typical ways to extract thin structures^31–33^. We use this as the first method to extract the PVSs from the original 7 T MR images or our enhanced and denoised images. In this method, 7 T MR image is first smoothed through a Gaussian kernel with a scale *s*, and then the eigenvalues of the second derivative matrix (Hessian matrix) of the smoothed image is computed. Note that the three eigenvalues (*λ*_1_, *λ*_2_, *λ*_3_) represent the magnitudes of derivatives for their associated eigenvectors *V*_1_, *V*_2_, *V*_3_, which represent three principal directions. If the smallest eigenvalue *λ*_1_ is small while the other two eigenvalues *λ*_2_ and *λ*_3_ are relatively large, a high vesselness can be obtained, and thus the respective voxel can be likely belonging to the tubular structure. More details on this vesselness measurement method can be found in Frangi *et al*.’s work^31^. In our application, we compute the vesselness values with two small scales (s = 0.5 and s = 1) for extracting very thin or relatively thick PVSs structures, and then use their maximum vesselness as the final vesselness for the respective voxel. Finally, we extract the voxels with higher vesselness than a certain threshold as PVSs. An example of vesselness map is shown in Fig. 6. We can identify a better vesselness map achieved using our enhanced and denoised image, as compared to the original 7 T MR image, especially for the zoom-up regions in the bottom-left corner of each subfigure in Fig. 6.

#### Random Forest Based Segmentation

We also apply a learning-based PVS segmentation method^16^ to our enhanced and denoised image. In the training stage, PVSs and non-PVSs voxels are sampled from a region of interest (ROI) determined by a certain vesselness threshold. Then, 3D randomized Haar features are extracted from those samples, and the sequential classifiers are learned using the random forest model^34–36^. To capture the consistent patterns of PVSs with different orientations and intensity inhomogeneity, both the principal directions and the intensity distribution of local region are normalized during the extraction procedure of the 3D randomized Haar features. In the testing stage, the Haar features are extracted at each target voxel in the ROI and are then passed through trained decision trees from the root node to a leaf node, with respect to the established decision functions of all selected nodes. All the class labels in the selected leaf nodes of all decision trees are averaged. The class label of the underlying voxel is finally determined by the maximal prediction.

## Experimental results

For quantitative comparison, we have created ground-truth labels for the 6 images and also for the right hemispheres of the remaining 11 images. We then verify the effectiveness of our enhancement method by comparing the PVS segmentation results achieved from (1) the original 7 T MR images, (2) the denoised images (by directly applying BM4D on the original 7 T MR images), and (3) our enhanced and denoised images (by our complete method). The segmentation performance is evaluated by the Dice similarity coefficient (DSC), sensitivity (SN), and positive prediction value (PPV) as defined below:

5 $$DSC=\frac{2TP}{2TP+FP+FN},\,\,\,\,\,SN=\frac{TP}{TP+FN},\,\,\,\,\,PPV=\frac{TP}{TP+FP}$$

where *TP*, *FP*, and *FN* denote the true positive, false positive, and false negative, respectively.

### Ground Truth

**Figure 7:**
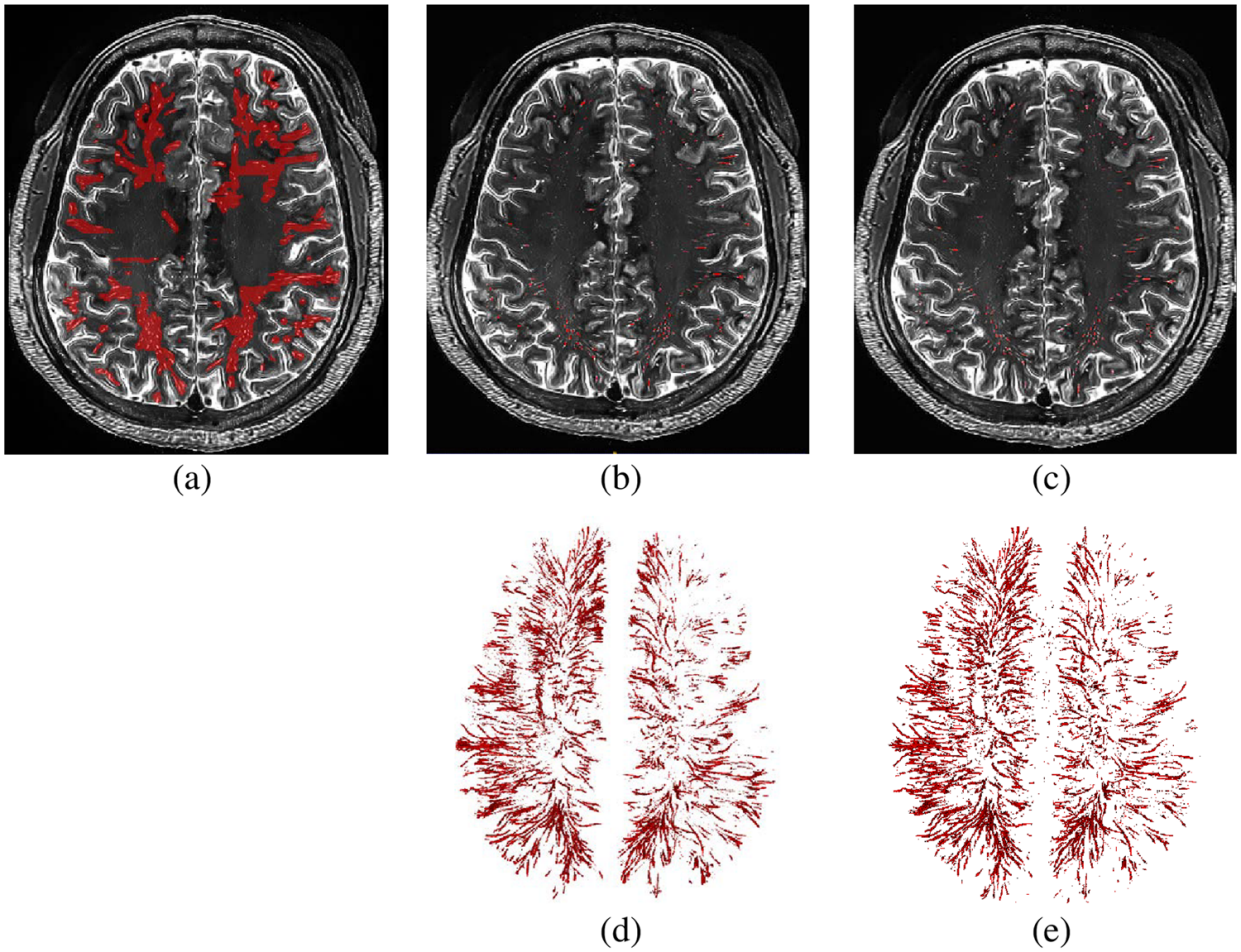
(**a**) The manually drawn ROIs of PVSs (red regions), (**b**) the ground-truth labels (red thin line structures) generated by intensity hard-thresholding upon the ROIs, followed by manual editing via two experts, (**c**) the same image with our PVS segmentation results (red thin line structures), (**d**) the 3D rendering of ground-truth labels, and (**e**) the 3D rendering of our PVS segmentation results.

The ground-truth labels were created by intensity-thresholding on a region of interest (ROI) defined manually. Note that it is very challenging to generate accurate PVSs ground-truth labels from the original images even though the ROI is carefully defined, since ambiguous noise is so close to the weak PVSs. On the other hand, in the enhanced images, there is less noise and the intensities of PVSs voxels are more distinguishable from the background voxels. Thus, we selected to generate the ground-truth labels from the enhanced images. Specifically, we manually marked the ROI that contains PVSs, and then extracted the voxels with higher intensities than a certain threshold as PVSs. Figure 7(a,b) show the manually drawn ROI (i.e., red voxels in Fig. 7(a)) and the ground-truth labels generated by the intensity-thresholding, followed by manual editing via two experts.

### Parameters

For the forward Haar transform of the nonlocal cubes, we extracted eight 7 × 7 × 7 cubes on a small neighborhood (3 × 3 × 3) of the reference cube, which was selected by the sliding window with the step of 7. As described in Section 2.2, we chose *T*_1_ and *T*_3_ as 50 and 150 so that the minimum and maximum Haar transform coefficients of PVSs could be included in this range, and then we chose *T*_2_ as 110 since most coefficients of PVS were included in the range between 110 and 150. The two parameters *γ*_1_ and *γ*_2_ for coefficient enhancement were heuristically set as 24 and 12, respectively, since coefficients of PVSs were sometimes around 50, while those of bright parts near the skull were around 1200. The final image denoising was performed by the BM4D method using the available code (http://www.cs.tut.fi/~foi/GCF-BM3D/) and their recommended settings^28^. The vesselness map was generated first by segmenting the white matter region with FAST^37^ and then by computing the eigenvalues of the Hessian matrix^31^ at each voxel within the white matter region. For the random forest based segmentation method, we conducted a leave-one-out cross-validation, i.e., testing an image by using the classifiers trained from all other 16 remaining images.

### PVS Segmentation by Vesselness Hard-thresholding

**Figure 8:**
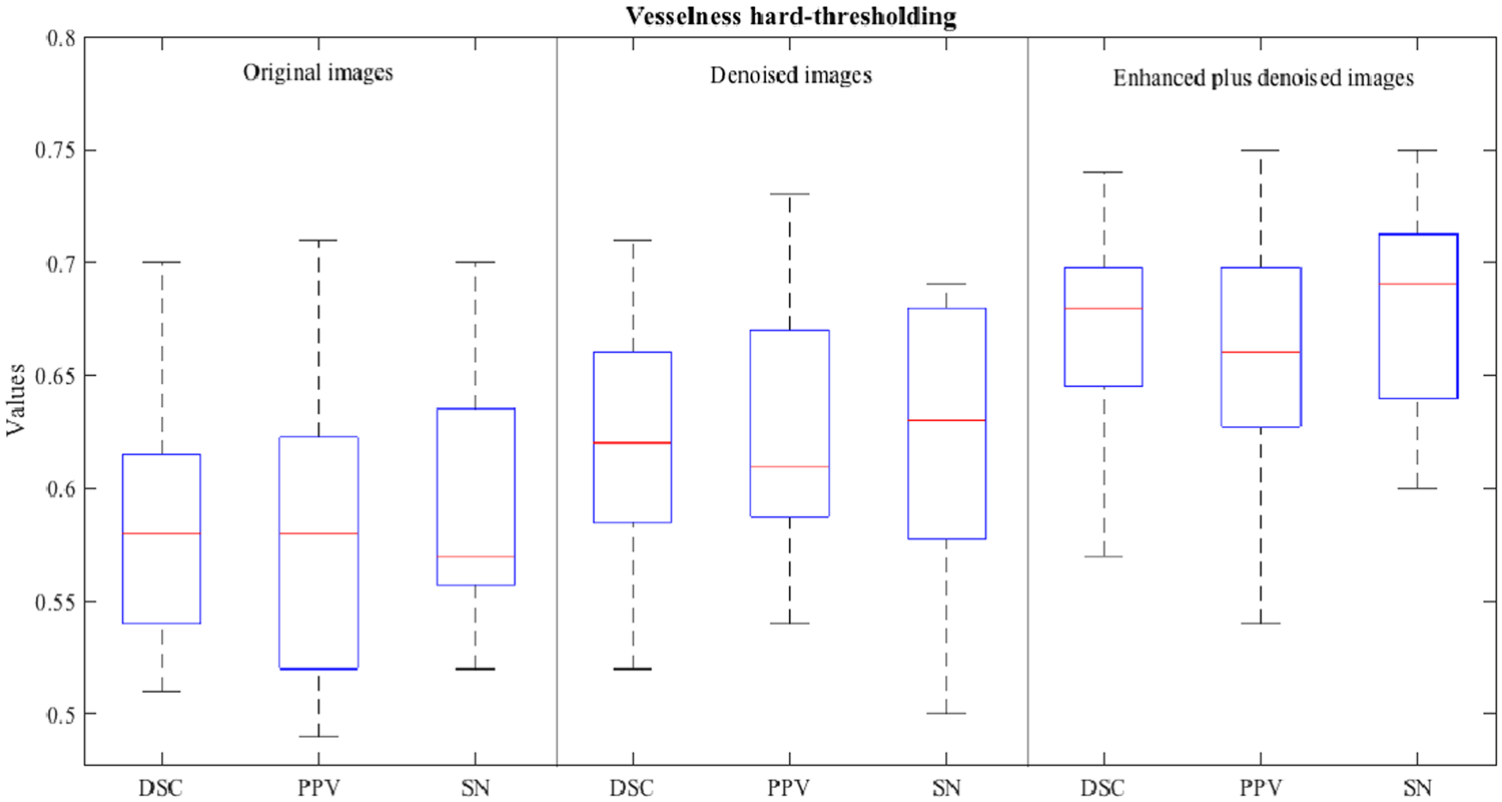
Segmentation accuracies of the vesselness hard-thresholding method for the original 7 T MR images (left), denoised images (middle), and our enhanced and denoised images (right).

In the vesselness thresholding method^16, 38^, the optimal vesselness threshold depends on the images under study. Thus, we tested several vesselness thresholds and then used the best one for each image. Table 1 show the segmentation accuracies for the three types of images. Figure 8 shows the box plots of the segmentation accuracies for the three types of images, i.e., (1) the original 7 T MR images, (2) the denoised images (by directly applying BM4D on the original 7 T MR images), and (3) our enhanced and denoised images (by our complete method). The average DSC score on the original 7 T MR images was only about 58%, because of too much ambiguous noise in the original images. When the original images were denoised by BM4D, the DSC score improved about 4%, and the highest DSC on our enhanced and denoised images could be achieved up to 67%. The PPV and SN scores present a similar situation. In summary, the best segmentation accuracies were achieved for the most cases when our enhanced and denoised images were used.

**Table 1:** Segmentation accuracy of the vesselness hard-thresholding method on the original 7 T MR images, the denoised images, and our enhanced and denoised images.

	Subj	1	2	3	4	5	6	7	8	9	10	11	12	13	14	15	16	17	Ave.
Original image	DSC PPV SN	0.61 0.68 0.56	0.59 0.61 0.57	0.55 0.58 0.52	0.55 0.56 0.54	0.54 0.52 0.57	0.57 0.55 0.60	0.58 0.61 0.56	0.51 0.49 0.53	0.63 0.62 0.63	0.67 0.66 0.67	0.61 0.58 0.65	0.53 0.51 0.56	0.70 0.71 0.70	0.60 0.63 0.57	0.64 0.62 0.66	0.52 0.49 0.55	0.54 0.52 0.57	**0.58 0.58 0.59**
Denoised image	DSC PPV SN	0.64 0.65 0.63	0.59 0.61 0.58	0.57 0.59 0.55	0.62 0.60 0.64	0.63 0.59 0.68	0.66 0.66 0.67	0.62 0.69 0.57	0.60 0.58 0.62	0.66 0.67 0.64	0.66 0.67 0.69	0.61 0.64 0.59	0.56 0.54 0.58	0.70 0.73 0.68	0.71 0.73 0.68	0.65 0.61 0.69	0.52 0.55 0.50	0.56 0.56 0.56	**0.62 0.63 0.62**
Proposed Enhanced image	DSC PPV SN	0.65 0.66 0.64	0.67 0.68 0.66	0.62 0.63 0.60	0.72 0.72 0.73	0.67 0.65 0.69	0.74 0.75 0.73	0.69 0.69 0.69	0.65 0.62 0.68	0.69 0.69 0.69	0.69 0.66 0.72	0.62 0.60 0.64	0.57 0.54 0.61	0.72 0.73 0.71	0.68 0.66 0.71	0.73 0.72 0.75	0.68 0.68 0.68	0.63 0.62 0.64	**0.67 0.66 0.68**

### PVS Segmentation by Random Forest

When we use the random forest for PVS segmentation, we need to design sequential classifiers for iteratively performing the segmentation. That is, the outputs of the first classifier are used as features to train the second classifier, along with the features already computed from the input image. This step continues until no performance can be improved with more classifiers. For the original 7 T MR image, we learned three sequential classifiers to achieve the best segmentation results. On the other hand, we found that, most noise was already removed in the denoised images (by directly applying BM4D on the original 7 T MR images) and our enhanced and denoised images, and thus the best results were achieved by using the first classifier (and using more classifiers did not improve the results) in these cases. Therefore, for each type of images, we used the best number of classifiers for segmentation.

**Figure 9:**
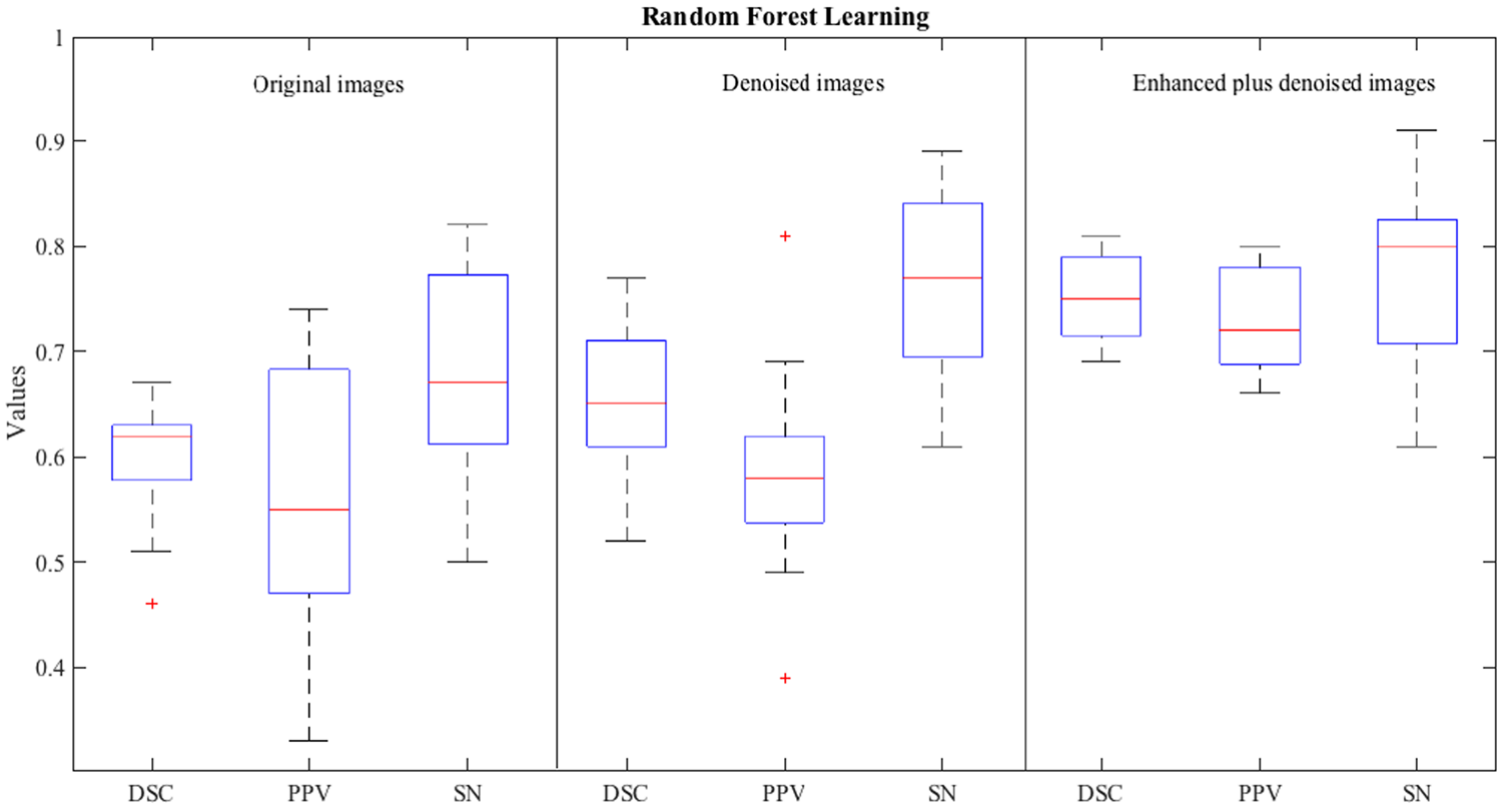
Segmentation accuracies of the random forest learning based method for the original 7 T MR images (left), denoised images (middle), and our enhanced and denoised images (right).

Experimental results are shown in Table 2. Figure 9 also shows the box plots of the segmentation accuracies for the three types of images. By comparing Table 2 with Table 1 and by comparing Fig. 9 with Fig. 8, we can see that, in most cases, the random forest based method can achieve much higher PVS segmentation accuracy than the hard-thresholding based method on every type of image. The highest segmentation accuracies were achieved when both our enhancement and denoising method (our complete method) and the random forest based method were used to segment PVSs. Figure 7(c,e) further shows an example of the predicted result by our complete method. As can be seen, our predicted PVSs are very similar to the ground-truth labels with the best PVS segmentation results, indicating that our proposed image enhancement and denoising method is helpful for PVS segmentation.

**Table 2:** Segmentation accuracy of the random forest method on the original 7 T MR images, the denoised images, and our enhanced and denoised images.

	Subj	1	2	3	4	5	6	7	8	9	10	11	12	13	14	15	16	17	Ave.
Original image	DSC PPV SN	0.65 0.61 0.69	0.62 0.52 0.77	0.52 0.47 0.59	0.61 0.68 0.55	0.67 0.70 0.64	0.67 0.69 0.65	0.60 0.74 0.50	0.62 0.74 0.53	0.62 0.63 0.62	0.59 0.47 0.82	0.51 0.38 0.75	0.46 0.33 0.78	0.62 0.54 0.82	0.63 0.55 0.74	0.63 0.51 0.80	0.63 0.64 0.62	0.54 0.46 0.67	**0.60 0.57 0.68**
Denoised image	DSC PPV SN	0.74 0.81 0.68	0.71 0.62 0.82	0.61 0.54 0.70	0.55 0.49 0.64	0.52 0.39 0.75	0.63 0.54 0.75	0.61 0.61 0.61	0.59 0.52 0.67	0.64 0.55 0.77	0.74 0.63 0.89	0.65 0.53 0.84	0.65 0.55 0.80	0.77 0.69 0.88	0.70 0.61 0.81	0.71 0.61 0.84	0.65 0.58 0.86	0.68 0.62 0.74	**0.66 0.58 0.77**
Proposed Enhanced image	DSC PPV SN	0.80 0.71 0.91	0.79 0.78 0.80	0.69 0.68 0.69	0.75 0.80 0.71	0.72 0.69 0.74	0.79 0.78 0.81	0.69 0.69 0.70	0.69 0.72 0.61	0.70 0.68 0.72	0.79 0.72 0.87	0.77 0.68 0.89	0.73 0.66 0.81	0.81 0.79 0.82	0.78 0.75 0.82	0.80 0.77 0.84	0.74 0.78 0.71	0.73 0.77 0.70	**0.75 0.73 0.77**

## Conclusion and Discussion

In this paper, we have presented a novel method for enhancing PVSs in the 7 T MR images. To demonstrate the effectiveness of our PVSs enhancement method, we have applied two existing PVS segmentation methods, i.e., (1) vesselness thresholding and (2) random forest learning, to the original 7 T MR images, denoised images, and our enhanced and denoised images, respectively. When the thresholding based method or the random forest method was directly applied to the original 7 T MR images without preprocessing, performance was limited due to very similar appearances of thin PVS and noise in the given images. On the other hand, the proposed preprocessing step effectively enlarged the gap between the appearances of thin PVS and noise, and thus the PVS segmentations from our enhanced and denoised images were much more accurate than those from the original and denoised images for both segmentation methods. The performance gains of the thresholding based method and the random forest method were 9% and 15%, respectively, while the random forest based method could achieve 8% higher average DSC accuracy than the thresholding based method. Specially, when using the vesselness thresholding based method, many errors occurred at the edges between white matter and gray matter, as the thin edges were very similar to the structures of PVSs. This issue can be effectively addressed using the random forest based method by utilizing contextual patterns of local neighbors. A potential limitation of our method is the heuristic setting of various thresholds and amplification factors. The intensity ranges of MR images depend on MR acquisition parameters, while the number of available images we have is only 17. Thus, it is hard to find the optimal parameters, since, even if the parameters are optimized, this can lead to overfitting. Accordingly, we left those parameters without parameter optimization. On the other hand, we believe that our released software package^29^ with multiple parameters would be able to provide more flexibility, when applied to other MR images acquired by different parameter settings. Regarding the PVS segmentation accuracy, some short and small structures were still observed occasionally in the segmented results, but most of the noise was removed in our enhanced and denoised image. In the future, we will further develop our method to remove those small structures (i.e., by using morphological filtering method^16, 38^) and also improve the overall performance of our complete method (i.e., by better coordinating the two enhancement and denoising steps in our method).
